# In Vitro Monitoring of Human T Cell Responses to Skin Sensitizing Chemicals—A Systematic Review

**DOI:** 10.3390/cells11010083

**Published:** 2021-12-28

**Authors:** Marina Aparicio-Soto, Caterina Curato, Franziska Riedel, Hermann-Josef Thierse, Andreas Luch, Katherina Siewert

**Affiliations:** 1 Department of Chemical and Product Safety, German Federal Institute for Risk Assessment, 10589 Berlin, Germany; Marina.Aparicio-Soto@bfr.bund.de (M.A.-S.); Caterina.Curato@bfr.bund.de (C.C.); Franziska.Riedel@bfr.bund.de (F.R.); Hermann-Josef.Thierse@bfr.bund.de (H.-J.T.); Andreas.Luch@bfr.bund.de (A.L.); 2 Institute of Pharmacy, Freie Universität Berlin, 14195 Berlin, Germany

**Keywords:** allergic contact dermatitis, chemical sensitizers, in vitro test, T cell assays, lymphocyte transformation test, antigen specificity

## Abstract

Background: Chemical allergies are T cell-mediated diseases that often manifest in the skin as allergic contact dermatitis (ACD). To prevent ACD on a public health scale and avoid elicitation reactions at the individual patient level, predictive and diagnostic tests, respectively, are indispensable. Currently, there is no validated in vitro T cell assay available. The main bottlenecks concern the inefficient generation of T cell epitopes and the detection of rare antigen-specific T cells. Methods: Here, we systematically review original experimental research papers describing T cell activation to chemical skin sensitizers. We focus our search on studies published in the PubMed and Scopus databases on non-metallic allergens in the last 20 years. Results: We identified 37 papers, among them 32 (86%) describing antigen-specific human T cell activation to 31 different chemical allergens. The remaining studies measured the general effects of chemical allergens on T cell function (five studies, 14%). Most antigen-specific studies used peripheral blood mononuclear cells (PBMC) as antigen-presenting cells (APC, 75%) and interrogated the blood T cell pool (91%). Depending on the individual chemical properties, T cell epitopes were generated either by direct administration into the culture medium (72%), separate modification of autologous APC (29%) or by use of hapten-modified model proteins (13%). Read-outs were mainly based on proliferation (91%), often combined with cytokine secretion (53%). The analysis of T cell clones offers additional opportunities to elucidate the mechanisms of epitope formation and cross-reactivity (13%). The best researched allergen was *p*-phenylenediamine (PPD, 12 studies, 38%). For this and some other allergens, stronger immune responses were observed in some allergic patients (15/31 chemicals, 48%), illustrating the in vivo relevance of the identified T cells while detection limits remain challenging in many cases. Interpretation: Our results illustrate current hardships and possible solutions to monitoring T cell responses to individual chemical skin sensitizers. The provided data can guide the further development of T cell assays to unfold their full predictive and diagnostic potential, including cross-reactivity assessments.

## 1. Introduction

Thousands of chemicals have a sensitizing capability [1,2]. In allergic individuals, skin exposure can trigger allergic contact dermatitis (ACD). Depending on the form of the chemical contact, respiratory, systemic and local symptoms at other body sites may occur [3]. In Europe, approximately 20–27% of the general population is allergic to at least one chemical allergen [4,5]. Nickel remains the most common sensitizer with an approximate prevalence of 11.4%, while reactions to fragrance mix I (3.5%), cobalt (2.7%), balsam of Peru (1.8%) and *p*-phenylenediamine (PPD, 1.5%) are also frequent [4]. Apart from metals, other important sensitizers comprise preservatives, drugs, excipients and many other substances of synthetic or natural origin [6,7,8].

Given the lack of causal therapies, reduced quality of life and even forced occupation changes, ACD constitutes a huge burden for personal and public health [9,10,11,12]. To tackle these challenges, accurate predictive and diagnostic tests are essential. Nowadays, the available predictive in vivo tests are limited by species differences and ethical considerations. In addition, the huge number of new compounds including nanomaterials that are constantly being developed by the chemical industry renders comprehensive in vivo testing impossible. Similarly, diagnostic epicutaneous patch testing has some disadvantages [13,14]. Patch testing may sensitize, although the risk is small for current standard substances [15] and boost existing allergies, at least locally [16]. Results can be unclear concerning distant skin eczema and patch testing may not be possible in patients with angry back syndrome or some other ongoing skin conditions [17]. For some allergens, suitable test substances are missing, or preparations do not penetrate the skin leading to false negative reactions, as demonstrated for PdCl_2_ or tattoo inks [18,19].

To overcome the shortcomings of in vivo tests, alternative in vitro tests have been developed and validated by the Organization of Economic Cooperation and Development (OECD). Established in vitro tests cover all pathogenic events of the adverse outcome pathway of skin sensitization, except for the final key event, which is T cell activation [20]. During the sensitization phase, chemical allergens bind proteins (key event 1), resulting in the activation of keratinocytes (key event 2) and dendritic cells (DCs, key event 3). DCs migrate to the draining lymph nodes and present chemical-induced epitopes to activate rare antigen-specific T cells (key event 4) among millions of irrelevant bystander T cells (≥10^8^ different T cell receptor (TCR) clonotypes per individual [21]). DC responses to chemical sensitizers critically determine T cell activation strength and subsequent effector and memory T cell responses, including tissue homing and subset formation with defined cytokine secretion capacities [22,23]. The function of DCs and other cells that may serve as antigen-presenting cells (APC) in the elicitation phase of ACD have been reviewed elsewhere and are also a matter of ongoing research [24,25,26,27,28,29,30]. Activated T cells proliferate, differentiate and distribute in the body, preferentially accumulating at tissue sites of previous inflammation as tissue-resident memory T cells (T_RM_) [16,31,32,33,34]. Subsequent encounters with the same allergen lead to the activation of powerful local antigen-specific T_RM_ cells and accompanying innate immune responses. After ~24 h, further (antigen-specific) memory T cells infiltrate from blood [27,35]. This relatively slow process of immune cell egress into the tissue is linked to the slow evolvement of clinical symptoms, thus the term delayed hypersensitivity. In addition, quick antibody-based effects or functions may play a minor role, depending on the experimental system [36].

Taken together, chemical-specific T cells are key players of allergic reactions, but in vitro detection has remained challenging [37]. Here, we review recent original research papers that succeeded in the detection of T cell activation to skin sensitizing chemicals. Since the main limiting step is unsecure epitope formation, we overview current knowledge in the following section.

### 1.1. Chemical-Induced T Cell Epitopes

**Figure 1:**
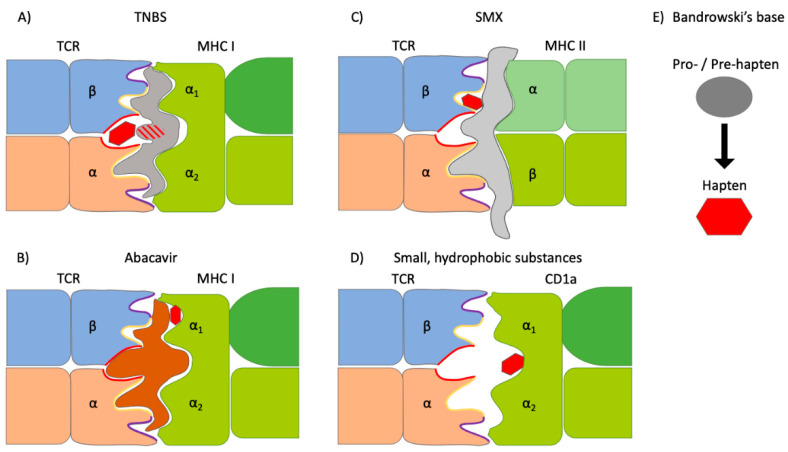
Mechanisms of T cell receptor (TCR) activation by non-metallic chemical allergens. (**A**) Chemical haptens (red trapeze) may bind covalently to major histocompatibility complex (MHC)-presented peptides (hapten concept). This has been shown for MHC I-restricted CD8+ T cells specific for the model chemical 2,4,6-trinitrobenzenesulphonic acid (TNBS) or the β-lactam antibiotic flucloxacillin. Murine responses seem to focus on a lysine modification at peptide position 4 (red-grey striped) [44,45]. (**B**) Some drugs associated with hypersensitivity reactions bind non-covalently, which is called pharmacological interaction (p-i) [46,47]. Binding via p-i has often been described in association with certain MHC alleles, termed human leukocyte antigens (HLAs) in humans (green). Abacavir, for example, binds to the F-pocket of HLA-B*57:01 resulting in the presentation of altered peptides (brown) [48,49]. (**C**) Some chemicals and metal ions form complexes at the TCR-pMHC interface. For sulfamethoxazole (SMX), binding to the complementarity-determining region 2 (CDR2) of TRVB-20-expressing TCR (blue) has been modeled [50]. (**D**) Haptens may displace endogenous lipid ligands on the MHC-like molecule cluster of differentiation (CD) 1a resulting in polyclonal αβ TCR activation to the CD1a surface [51]. (**E**) Pro- or pre-haptens require auto-oxidation or processing by metabolizing enzymes to become protein-binding.

Much progress has been made in the understanding of metal-induced T cell epitopes [38,39,40,41], which has been reviewed elsewhere [42,43]. Mechanisms of non-metallic chemical-induced T cell epitopes, including those of drug hypersensitivity reactions (DHRs), are illustrated in Figure 1.

TCRs recognize cognate peptides (p) presented by proteins of the major histocompatibility complex (MHC), also called human leukocyte antigens (HLAs) in humans [52]. Self TCR-pMHC complexes are usually ignored by the immune system due to negative selection in the thymus. In the case of chemical allergens, modified self-structures exceed the threshold for functional T cell binding and induce unintended adaptive immune responses. These mechanisms are grounded in the extensive poly-specificity (also called cross-reactivity) of TCR [43,53,54].

Chemical sensitizers may bind covalently to proteins, a process termed haptenization. Recognition of a covalently bound chemical on MHC-presented peptides by T cells was first shown using the model chemical 2,4,6-trinitrobenzenesulphonic acid (TNBS, Figure 1A) [55]. TNBS generates antigenic trinitrophenyl (TNP) determinants. TNP-modified peptides may replace unmodified peptides on MHC proteins on the surface of APC [55]. Another option is a short-term TNBS modification of APC, which leads to the binding of chemicals to surface pMHC [56,57,58].

However, most often, haptens are thought to modify extracellular proteins, which afterwards are incorporated and processed by APC leading to the presentation of haptenated peptides on MHC proteins. If the hapten enters the cell, intracellular proteins may get modified. In addition, haptens may influence antigen processing, leading to the presentation of cryptic epitopes by MHC proteins that do not contain the chemical [59].

In mice, TNBS-specific H-2K^b^-(MHC I)-restricted CD8+ T cells have unusually high frequencies [60,61,62]. The underlying mechanism seems to be a carrier peptide-independent recognition of TNP-modified free ε-amino groups of lysine residues at peptide position (p) 4 by many different TCR [44]. In addition, lysine at p7 may get TNP-modified, but T cells recognize this structure only in the context of a unique peptide and less frequently. Thus, the role of the MHC-presented peptide can vary in chemical-specific T cell recognition and this supposedly has to be individually assessed for each epitope. So far, a common gene segment use among TNBS-specific T cells has been suggested but not confirmed [62,63].

Among relevant human sensitizers, β-lactam antibiotics have been shown to act via covalent binding. The classic example for covalent binding drugs is penicillin G [64]. Another interesting example is flucloxacillin, for which hypersensitivity is strongly associated with HLA-B*57:01. Patient-derived T cells mainly recognize a covalently modified peptide [65,66]. In mice, hypersensitivity could be induced with a peptide modified at a p4 lysine residue [45].

However, flucloxacillin may also bind non-covalently, which is the major recognition mechanism for in vitro T cell activation in non-allergic HLA-B*57:01-expressing individuals [67]. The direct and reversible interaction of drugs with the HLA or the peptide in a non-covalent manner is termed pharmacological interaction (p-i) with immune receptors (Figure 1B) [46,47]. Flucloxacillin activity dependent on high drug concentrations was independent of proteasomal processing and immediate, indicating direct binding to the TCR-pMHC interface [67]. A third mechanism for flucloxacillin T cell epitope formation was recently shown, which involves the binding in the peptide-anchoring pockets of HLA-B*57:01 and the presentation of an altered peptide repertoire (Figure 1B) [45]. In summary, the flucloxacillin case demonstrates the importance of patient analysis to determine the in vivo relevance of different epitope formation mechanisms.

Binding via p-i has often been reported in the context of HLA allele-associated drug hypersensitivities [68,69,70,71,72,73,74]. Arguably, the most prominent example is abacavir binding to the F-pocket of HLA-B*57:01, which conceals a carboxy-terminal tryptophan important for peptide anchoring. The shape of the antigen-binding cleft changes upon abacavir binding, resulting in the presentation of an altered peptide repertoire [48,49]. This activates neo-antigen-specific CD8+ T cells in patients [75]. In all mentioned cases of HLA allele-associated binding, the TCR has no direct chemical contact.

Non-covalent interactions with direct chemical TCR contact may involve binding to the MHC outside of the peptide-anchoring pockets, to the presented peptide or to the TCR. TCR binding was modeled using molecular dynamic simulation for a TRBV-20-expressing sulfamethoxazole (SMX)-specific TCR (Figure 1C). Here, the TCR binds to SMX with high affinity through the conserved β-chain complementarity-determining region (CDR) 2 domain. SMX binds via TYRβ57, ASPβ64 and LYSβ65, which in the unbound TCR are responsible for hydrogen bonds to adjacent CDR loops. Therefore, the overall TCR conformation is changed, although a functional link to the allergic reaction remains missing [50].

Recently, a new mechanism of CD1a-restricted chemical-specific T cell activation has been described (Figure 1D) [51]. Several skin cells express CD1a proteins that accommodate endogenous lipid ligands which interfere with the activation of autoreactive CD1a-specific T cells [76,77]. Autoreactive T cells constitute ~1% of the skin T cell pool. Chemical sensitizers such as farnesol displace the endogenous ligands, then the TCR has direct contact with the unliganded surface which provokes autoreactive T cell responses. Alternatively, some chemicals may induce de novo lipid presentation on CD1a in certain APC, which may also activate T cells [78].

PPD, one of the most frequent skin sensitizers, binds non-covalently via a p-i mechanism, but as a pro-hapten, requires prior autoxidation (Figure 1E) [79,80]. Bandrowski’s base (BB), a trimeric autoxidation product of PPD, is a pre-hapten requiring cellular metabolism to form T cell epitopes [80].

The variety in chemical reactivity mechanisms and the many different possible target proteins make it difficult to predict T cell epitopes [81,82]. In addition, rare epitopes can be important since T cells can be activated by single ligands [83,84]. While experimental research on the haptenome of sensitizing chemicals is ongoing [85,86], new insights into possible T cell epitopes are obtained that need to be experimentally validated, e.g., as outlined in the studies reviewed here.

### 1.2. Review Objectives

In the present review, we systematically review the available literature on in vitro T cell activation achieved with non-metallic chemical allergens in the last 20 years. We focus on skin-sensitizing substances, since these represent one of the most relevant groups of sensitizers on a general population scale. The results and general principles for in vitro T cell activation can be transferred to any sensitizing chemical. Our results aim to provide directions for further attempts on the establishing of in vitro T cell assays for sensitizing chemicals, which are crucial for the further development of predictive and diagnostic tests.

## 2. Methods

### 2.1. Search Strategy

The present review was conducted in accordance with the Preferred Reporting Items for Systematic Reviews and Meta-Analyses (PRISMA 2020 statement) [87]. Three screeners (MAS, CC and KS) designed a search strategy including articles indexed and published in the last 20 years (2001–2021) in PubMed and Scopus. We included the following criteria of interest as keywords (see also Supplementary Materials, Table S1).

#### 2.1.1. PubMed

“t-lymphocytes”[MeSH Terms] AND ((“2001/01/01 00:00”:”3000/01/01 05:00”[Date—Publication] AND “journal article”[Publication Type]) NOT “review”[Publication Type]) AND ((“dermatitis, allergic contact”[MeSH Terms] OR “chemical allergen”[Title/Abstract] OR “chemical allergens”[Title/Abstract] OR (“hypersensitivity”[Title/Abstract] AND “dermatitis”[Title/Abstract])) AND ((“2001/01/01 00:00”:”3000/01/01 05:00”[Date—Publication] AND “journal article”[Publication Type]) NOT “review”[Publication Type])) AND “English”[Language] AND (“human s”[All Fields] OR “humans”[MeSH Terms] OR “humans”[All Fields] OR “human”[All Fields]).

#### 2.1.2. Scopus

((TITLE-ABS-KEY(T cell) OR TITLE-ABS-KEY(T cells) OR TITLE-ABS-KEY(T-cell) OR TITLE-ABS-KEY(T-cells) OR TITLE-ABS-KEY(T lymphocyte) OR TITLE-ABS-KEY(T lymphocytes) OR TITLE-ABS-KEY(T-lymphocyte) OR TITLE-ABS-KEY(T-lymphocytes)) AND (TITLE-ABS-KEY(allergic contact dermatitis) OR TITLE-ABS-KEY(contact allergy) OR TITLE-ABS-KEY(contact dermatitis) OR (TITLE-ABS-KEY(hypersensitivity) AND TITLE-ABS-KEY(dermatitis)) AND TITLE-ABS-KEY(human)) AND TITLE-ABS-KEY (in vitro)) AND (LIMIT-TO (PUBYEAR,2021) OR LIMIT-TO (PUBYEAR,2020) OR LIMIT-TO (PUBYEAR,2019) OR LIMIT-TO (PUBYEAR,2018) OR LIMIT-TO (PUBYEAR,2017) OR LIMIT-TO (PUBYEAR,2016) OR LIMIT-TO (PUBYEAR,2015) OR LIMIT-TO (PUBYEAR,2014) OR LIMIT-TO (PUBYEAR,2013) OR LIMIT-TO (PUBYEAR,2012) OR LIMIT-TO (PUBYEAR,2011) OR LIMIT-TO (PUBYEAR,2010) OR LIMIT-TO (PUBYEAR,2009) OR LIMIT-TO (PUBYEAR,2008) OR LIMIT-TO (PUBYEAR,2007) OR LIMIT-TO (PUBYEAR,2006) OR LIMIT-TO (PUBYEAR,2005) OR LIMIT-TO (PUBYEAR,2004) OR LIMIT-TO (PUBYEAR,2003) OR LIMIT-TO (PUBYEAR,2002)) AND (LIMIT-TO (LANGUAGE,”English”)) AND (LIMIT-TO (DOCTYPE,”ar”)).

### 2.2. Inclusion and Exclusion Criteria

We included only original articles written in English language available in a full-text form from 2001 to 2021 (date of the search: 27 September 2021). The following inclusion criteria were used: (i) in vitro studies using chemicals involved in ACD, (ii) studies investigating in vitro human T cell activation to non-metallic chemical allergens.

We did not consider: (i) reviews, (ii) book chapters, (iii) protocols, (iv) editorials/comments/opinions, (v) publications in languages other than English, (vi) duplicates (articles found in more than one database), (vii) conferences papers, (viii) letters/communications, (ix) articles that did not analyze in vitro human T cell activation upon contact with non-metal chemical allergens and (x) immune-histochemical studies of skin biopsies without further analysis of in vitro T cell activation.

### 2.3. Data Extraction and Collection

MAS and CC independently revised the articles identified by the search and evaluated whether they met the eligibility criteria to be included in this review. Potential disagreements were resolved through critical discussion with KS. All potentially relevant publications were retrieved in full. In addition, other relevant or up-to-date publications in the field have been included in the introduction and discussion sections.

### 2.4. Scoring System for Antigen-Specific T Cell Activation

We employed a scoring system to account for the varying degree of experimental evidence obtained for T cell activation to individual chemical allergens. MAS, CC and KS independently assigned a score (+++, ++, +) and the final score was decided on by common agreement. The highest score (+++) was given to chemicals for which multiple independent studies showed antigen-specific T cell activation. A medium degree of experimental evidence was labeled ++ and comprised chemicals that were investigated in at least two independent studies or that were associated with additional confirmation, e.g., by re-stimulation of T cell clones. The remaining chemicals from studies reporting antigen-specific T cell activation were graded +.

## 3. Results

### 3.1. Selection of Articles Following PRISMA Guidelines

**Figure 2:**
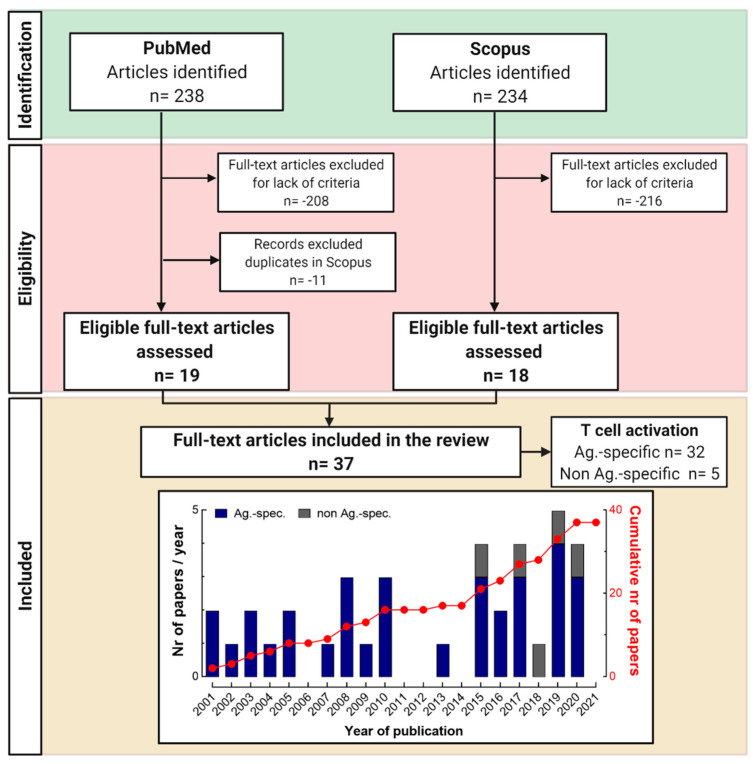
Flowchart of the search strategy applied in this systematic review according to the PRISMA statement 2020 guidelines [87]. The stacked bar histogram represents the time distribution of the articles included by year of publication (antigen (Ag.)-specific, blue, vs. non Ag.-specific, grey) and the red line the cumulative number of papers over the last 20 years (2001–2021).

We conducted searches in the PubMed and Scopus databases, following the strategies described in the methods (Section 2.1, Section 2.2 and Section 2.3). All original research articles published between 2001 and 2021 describing the in vitro activation of human T cells by non-metallic chemical allergens in the context of ACD were identified (Figure 2). We identified 238 and 234 publications, respectively. After the screening of the selected articles in PubMed, 208 articles were not included due to a lack of eligibility (see Section 2.2), 11 articles were duplicated in the Scopus database and 19 full-text articles were included in the review. Among the 234 articles obtained in the Scopus database, 216 were excluded because of a lack of criteria (see Section 2.2), leaving 18 records for screening. In total, we reviewed 37 articles and referred to them here with first author and publication year in addition to the bibliography numbering system. Among these, 32 publications described antigen-specific T cell activation (17 from PubMed, 15 from Scopus) and the others non-TCR-mediated T cell activation (2 from PubMed, 3 from Scopus) [88,89,90,91,92].

### 3.2. Monitoring Chemical-Specific T Cell Responses In Vitro

#### 3.2.1. Investigated Chemical Allergens

From the 32 papers on antigen-specific T cell activation, we identified T cell responses to 31 chemical skin sensitizers (Table 1). Among them, 28 chemicals were of human relevance, including fragrances (12), drugs (8), hair dyes and dye derivatives (2) and 6 other compounds, e.g., plant derivatives, preservatives and pollutants. Additionally, studies described T cell activation to model chemicals ((2,4-Dinitrobenzenesulfoniacid (DNBS), 2,4-Dinitrochlorobenzene (DNCB) and 1-Fluoro-2,4-dinitrobenzene (DNFB)).

To reflect the different experimental evidence obtained for the various chemicals on antigen-specific T cell activation, we applied a score (see Section 2.4). Besides the number of studies that independently assessed T cell activation, we also considered additional experiments, e.g., re-stimulation of T cell clones.

The most researched allergen was PPD, which was investigated in 12 independent studies (38%). BB, a trimeric product of PPD, was investigated together with PPD in four studies (13%). Both chemicals were assigned a +++ score regarding their ability to detect TCR-mediated T cell activation. A few sensitizers were investigated in at least two independent studies or T cell activation was additionally confirmed, e.g., by re-stimulation of T cell clones. These chemicals were assigned a ++ score (e.g., benzyl cinnamate, eugenol, methylchloroisothiazolinone/methylisothiazolinone (MCI/MI)). For the remaining chemicals, results were retrieved from only one study or one experimental evidence and a + score was assigned. The following paragraphs will provide more details on the experimental details in the individual studies (summarized in Supplementary Material, Table S2).

#### 3.2.2. Approaches for Chemical-Induced T Cell Epitope Formation

Different APC and epitope generation strategies were used by the different studies to observe antigen-specific T cell activation in vitro. Table 2 summarizes the choice of APC and the method of chemical administration.

The majority of the experimental attempts used PBMC-derived cells (24/32 studies, 75%), which contain all cell types, i.e., APC such as monocytes and B cells and all circulating T cell subsets. Alternatively, monocyte-derived dendritic cells (MoDCs, 6/32 studies, 19%) or Epstein–Barr Virus (EBV)-transformed B cells were used (3/32 studies, 9.4%). The possibility to observe antigen-specific T cell activation is critically dependent on the use of autologous APC except for antigens presented by the conserved MHC I-related molecule CD1a, which may be investigated using monocyte-like cell lines, e.g., K562 as APC (3/32 studies, 9.4%).

Most studies relied on a direct administration of the chemical of interest to the cell culture media to generate allergen-induced T cell epitopes in vitro (23/32 studies, 72%). In nine studies (29%), APC were pulsed with the chemical allergen from 10 min up to 24 h, then washed and co-cultured with T cells. We encountered four publications (13%) where the chemicals (i.e., MI and PPD) were presented as a protein conjugate, i.e., coupled to human serum albumin (HSA).

A practice to determine a non-toxic chemical concentration (e.g., by testing cell viability) before measuring T cell activity upon chemical exposure was used by five studies (16%). All chemical concentrations are listed in Table S2 (Supplementary Material). Protein-conjugated chemicals (i.e., PPD- and MI-HSA) induced a comparable or even better proliferative response compared to the soluble correspondent chemical (Supplementary Material, Table S2) (Jenkinson, 2010; Oakes, 2017; Popple, 2016; Wicks, 2019) [79,100,102,116]. Jenkinson and colleagues (2010) [79] calculated the equivalent molar scale of soluble and HSA-associated PPD to compare the strength of induced activation/proliferation. They revealed that HSA-bound PPD possesses a stronger antigenic capacity. In the case of the protein-bound forms of the chemicals, the induced proliferative responses follow the classical dose-dependent trend and better correlate to patients’ patch test results (Popple, 2016; Wicks, 2019) [102,116]. Soluble chemicals generally become toxic at higher concentrations. Notably, a baseline proliferation response to HSA may be taken into account. Soluble MI and HSA alone induce T cell proliferation in 7 and 9 patients out of 31, respectively, while 17/31 patients responded to MI-HSA (Popple, 2016) [116].

#### 3.2.3. Blood as Major T Cell Source

Almost all screened publications (29/32 studies, 91%) relied on PBMC as the source for T cells. Three publications (9.4%) presented results obtained in T cell-like cell lines that sometimes expressed a single TCR. One study studied T cell clones derived from patch test skin lesions in parallel to PBMC (Newell, 2013) [105].

Eight studies (25%) investigated the contribution of the two main CD4+ and CD8+ T lymphocyte subsets (except for one study focusing on CD4+ memory T cells (Kim, 2016) [120]). Three publications explored the involvement of naïve and/or memory T cell subsets (Gibson, 2015; Kim, 2016; Li, 2019) [94,119,120]. Two publications studied cell frequencies of CD4+ and CD8+ naïve and memory T cells (Oakes, 2017; Wicks, 2019) [100,102].

#### 3.2.4. Detection of Chemical-Specific T Cell Activation (Read-Outs)

The read-outs used in the reviewed articles to observe antigen-specific T cell activation in vitro are listed in Table 3. Cellular proliferation was the most frequent read-out (27/32 studies, 91%), measured by thymidine incorporation (21/32 studies, 66%), carboxy fluorescein diacetate succinimidyl ester (CFSE) dilution (2/32 studies, 6.2%) or other methods (4/32 studies, 13%). One study directly assessed the frequencies of antigen-specific T cells by ex vivo enzyme-linked immune-spot (ELISpot) assay (Newell, 2013) [103].

Seventeen studies (53%) measured secretion of inflammatory and/or T_H_-subset-specific cytokines (e.g., IL-4, IL-5, IL-17A, IFN-γ) by enzyme-linked immuno-sorbent assay (ELISA) (11/32 studies, 34%), ELISpot (5/32 studies, 16%) or other methods (e.g., intracellular staining, 3/32 studies, 9.4%) following a few days of cellular expansion. In 9 out of these 17 studies, proliferation was measured in parallel (marked with ** in Table 3). We observed a trend for a preferential differentiation towards the T_H_2 lineage in the cytokine production (5/17 studies, 29%) for PPD (3/17 studies, 18%) (Coulter, 2010; Jenkinson, 2010; Sieben, 2002) [79,80,93] and MCI/MI (1/17 studies, 6%) (Masjedi, 2003) [107]. Two studies defined a T_H_1 cytokine profile of chemical-specific T cells, i.e., for DNCB (note: T_H_2 shift in atopic patients) (Newell, 2013) [103] and fragrances (Sieben, 2001) [105]. In three studies (18%), chemical-stimulated cells secreted a mix of T_H_1 (e.g., IFN-γ) and T_H_2 (e.g., IL-4, IL-5 and/or IL-13) cytokines. Chemicals utilized in these three studies partially overlapped with the ones mentioned above as inducing a T_H_2 profile, i.e., PPD, BB, MCI, fragrance mix and parthenolide (Gibson, 2015; Moed, 2005; Wahlkvist, 2008) [94,95,117]. The remaining eight studies (47%) did not measure a conclusive, in this regard, panel of cytokines (e.g., IL-1α/IL-1β or IFN-γ/TNF-α/IL-2 or IFN-γ alone).

Gene expression by real-time quantitative polymerase chain reaction (RT-PCR), microarray or RNA sequencing (4/32 studies, 13%) and cellular phenotype/activation changes (e.g., CD69 expression by flow cytometry, 5/32 studies, 16%) were frequent additional read-outs, especially among more recent publications (Table 3, Supplementary Material Table S2).

None of the studies made conclusive observations on major differences in the activation or role of CD4+ and CD8+ T cell subsets in chemical-associated allergies. Sieben and colleagues (2001) [105] observed that 83% of established eugenol-specific T cell clones were CD4+HLA-DR+, and the remaining 17% were CD8+. Wicks, 2019 [102] and Oakes, 2017 [100] both observed a shift from the central memory (CM) to the effector memory (EM) compartment in PPD and PPD-HSA stimulated CD4+ and CD8+ T cells of allergic patients. Additionally, in the former study, an expansion of naïve T cells was detected in the blood compartment. A simultaneous contraction of the memory T cell population (probably due to recruitment to the site of patch test application) was also observed [102].

Four studies (13%) nailed antigen-specific T cell involvement by generating T cell clones confirming their proliferative ability upon re-stimulation with the original antigens, PPD and BB (Gibson, 2015; Jenkinson, 2010; Sieben, 2002; Skazik, 2008) [79,80,94,101]. Two studies performed HLA-blocking during T cell clone re-stimulation to confirm MHC-restricted T cell activation (Kim, 2020; Sieben, 2002) [80,108].

TCR features were addressed in two PPD-related studies (Oakes, 2017; Skazik, 2008) [100,101]. Oakes, 2017 [100] performed an unbiased high-throughput sequencing of the TCR α- and β-chains of PBMC derived from one PPD-allergic patient in ex vivo conditions after 6 days of culture with PPD-HSA. Approximately 800 TCR α- and β- chain sequences (0.8% of all detected TCR) were considered PPD-specific due to their increased frequencies compared to controls. A skewed V- and J-gene segment usage was observed while a mechanistic association with PPD recognition remains to be defined. The study by Skazik, 2008 [101] showed by flow cytometry that 8 out of 21 PPD-specific T cell clones expressed TRBV14 (Vβ16 in Arden nomenclature), a segment not highlighted in the study of Oakes, 2017 [100].

#### 3.2.5. Features of Chemical-Specific T Cell Responses in Patients

Studies varied in terms of patients’ cohort composition and experimental setups. Four case reports (13%) included only one to two patients with drug allergies (Girardi, 2015; Kim, 2020; Sachs, 2001; Vilchez-Sánchez, 2020) [108,111,112,113]. The remaining articles included cohorts with approximately 10 and up to 200 patch tested allergic patients. The proliferative response of allergic patients’ T cells to chemicals showed great variability. Generally, cells derived from patients with a very strong (+++) result in patch tests reacted more often and possessed a higher proliferative response than cells from patients with strong (++) or weak (+) patch test results. A general observation on the existence of a concordance between the patient patch test result and the patient T cell proliferative or cytokine response in vitro has been made by 4 out of 32 studies (13%) in the case of PPD (Bordignon, 2015; Wicks, 2019) [96,102], MCI/MI (Masjedi, 2003) [107] and parthenolide (Wahlkvist, 2008) [117]. Of note, three studies did not confirm this concordance for PPD (Moed, 2005) [95], MI (Popple, 2016) [116] and various fragrances (Sieben, 2001) [105]. Three studies (9.4%) tested the detection of chemical-specific T cells after administration of a cytokine cocktail (e.g., IL-7 + IL-12 or IL-4) to the culture media (Kneilling, 2009; Moed, 2005; Schutte, 2019) [95,99,104]. The addition of cytokines may support the proliferative capacity of chemical-specific T cells.

PPD and its derivative BB were investigated for potential T cell cross-reactivity (2/32 studies, 6.3%). For this purpose, Gibson, 2015 [94] and Sieben, 2002 [80] tested PPD- and BB-specific T cell clones from allergic and healthy donors. Gibson et al. found that 75% of PPD-specific T cell clones reacted exclusively to the original antigen, while Sieben et al. found that most of the 25 PPD-specific T cell clones were BB cross-reactive. Of note, BB-specific T cell responses are observed in all individuals, but PPD-specific T cells have been described only in allergic patients (Coulter, 2010; Gibson, 2015; Sieben, 2002) [80,93,94].

### 3.3. Monitoring Non-Antigen-Specific T Cell Activation

Five studies assessed T cell responses to chemical sensitizers (42 substances) in a non-antigen-specific manner (Supplementary Material, Table S3). Most chemicals were fragrance agents (13), drugs (11), dyes (5) and model chemicals (3), apart from preservatives, disinfectants and some industrial agents.

Frombach, 2018 [88] assessed immunotoxic influences of chemicals on cytokine secretion as well as IL-23R/CD119, CD124 and CD44 surface expression on expanded T cells derived from mixed lymphocyte reactions containing MoDC, T cells and allogenic keratinocytes. Similarly, Clouet, 2019 [92] monitored T cell proliferation in a mixed-lymphocyte reaction with THP-1 as a DC model. The increase in co-stimulatory capacity by sensitizer-treated DC reflects their potential to support antigen-specific T cell proliferation.

Hou, 2020 [89] used the Jurkat T cell line to measure increased CD69 expression upon exposure to 24 non-metallic sensitizing chemicals compared to control substances (Supplementary Material, Table S3). This approach is reminiscent of systems that assess activation of keratinocytes or DC by sensitizing chemicals [121,122]. While the authors hypothesize that Jurkat T cells may present chemical-induced epitopes, the recognition of this diverse chemical set by the only TCR that Jurkat cells express has not been backed-up by additional experiments.

Baló-Banga, 2015 [90] measured increased IL-6 levels in PBMC cultures from individuals with suspected immediate or delayed drug hypersensitivities 20 min after drug exposure [90]. The cellular IL-6 source, as well as the mechanism of its release, remain to be determined. Mai, 2017 [91] identified increased levels of T_H_17- and T_H_22-producing T cell subsets in polyclonal stimulated PBMC from formaldehyde-exposed workers with ACD history, indicating the outgrowth of the respective T cell subsets [91].

## 4. Discussion

In recent years, TCR-mediated in vitro T cell activation has been detected to a number of chemical allergens. Here, we present possible experimental solutions to the unique challenge of chemical-induced epitope formations. We link chemical identities and methodological details with the possibility to detect chemical-specific T cells.

### 4.1. APC Choice

A multitude of cells have been used as APC for in vitro T cell assays. The reviewed studies mainly employed PBMC and PBMC-derived DC such as MoDC or EBV-transformed B cells (Table 2). In the literature, the use of skin-derived APC such as Langerhans cells (LCs) or fibroblasts has also been described but this APC source is hardly available since it requires scarce autologous skin tissue [123].

EBV-transformed B cells are an intriguing source of APC since they can be propagated limitlessly, e.g., for clone re-stimulation. However, it takes a few weeks to generate EBV-transformed cells and requires a biosafety level 2 lab [94]. HLA-deficient cell-lines transfected with the HLA molecule of interest constitute a further APC option restricted to chemicals for which an HLA association has been identified. Once T cell clones have been established, they usually express MHC II and some can be stimulated without further APC, likely depending on the presented antigen peptide [39]. A few chemical-specific T cell clones tolerant to the HLA haplotype or acting MHC independently have been described [124]. This observation certainly does not warrant a general use of allogenic APC since mixed-lymphocyte reactions usually superimpose any antigen-specific signals. However, CD1a-reactive T cells can be studied using CD1a-transfected cell lines.

Some T cell populations require the presence of specialized APC. For instance, naïve T cells only proliferate upon contact with professional APC such as MoDC [56]. In addition, some chemical-specific T cell clones depend on tissue-restricted epitopes that are not presented by other APC, e.g., PBMC [78,123]. Thus, PBMC-based assays may not capture the complete chemical-reactive T cell pool but probably detect enough representative T cells to allow sound scientific conclusions. In case of pre-haptens, the choice of APC may influence metabolisms and thus epitope formation. None of the reviewed studies compared T cell responses using different APC. Of note, a high-enough APC density is mandatory to ensure efficient in vitro T cell contact and successful T cell activation [39].

### 4.2. T Cell Epitope Formation

The most critical step of in vitro T cell assays that investigate chemical allergens is the adequate formation of chemical-induced T cell epitopes. Protein antigen-specific T cells have been detected with frequencies as low as 1 in 10^7^ using enrichment methods and a sufficient number of input cells [125]. Thus, techniques are available to interrogate virus-specific cross-reactive T cell memory or the antigen-specific naïve T cell pool [126]. However, if chemical-induced epitopes are formed inefficiently and if this is combined with the rarity of antigen-specific T cells, the detection of T cell activation may become virtually impossible. In addition, epitopes may form in an HLA allele-restricted manner, which is less well investigated for sensitizing chemicals that are not used as drugs [68,69,70,71,72].

The knowledge on T cell epitope identity and the conditions needed for an efficient generation remains very limited and it has to be experimentally determined. Incubation time and chemical concentration are important determinants, as well as temperature and pH value, in order to mimic physiological conditions. In general, three major methods for epitope generation can be distinguished: (i) direct administration of chemicals into the APC–T cell co-culture, (ii) a separate chemical modification of APC and posterior addition to the T cell culture and (iii) allergen-modification of model proteins or peptides as an antigen source.

For haptens that form epitopes directly via covalent binding, APC modification with a high chemical concentration for a short time (e.g., 10–15 min at 37 °C) in PBS seems the most efficient epitope generation method as shown for the model allergens TNBS, DNBS or fluorescein isothiocyanate (FITC) [56,110,127]. This short-term modification method is not suitable for pre- or pro-haptens. Thus, a loss in epitope formation efficiency is expected if the active hapten is only formed during longer culture periods. Variations in experimental conditions, e.g., the addition of a cytochrome P450 cocktail or the antioxidant glutathione may help to evaluate whether a chemical acts as pre- or pro-hapten [80]. In addition, APC fixation or measurements on the timing of T cell responses (Ca^2+^ influx) can inform on the necessity for antigen processing and HLA block on the MHC restriction in experiments using bulk T cell cultures or T cell clones.

For chemicals that bind via a p-i mechanism, the binding affinity decides whether pre-incubated, washed APC, i.e., close to zero concentrations of the free chemical, can be used to detect T cell activation. Abacavir has a high affinity to HLA-B*57:01, so washed APC have been employed [48].

Most commonly, chemicals are directly added to the APC–T cell co-culture (Table 2). Here, toxic effects restrict the use of high chemical concentrations while frequencies of reactive T cells often correlate with the amount of the chemical present in the culture [39,67,128]. The use of rather high (albeit non-toxic) chemical concentrations likely enables the detection of the complete reactive T cell pool. However, in the case of flucloxacillin, in vitro T cell responses to high chemical concentrations observed in non-allergic individuals (processing-independent p-i mechanism) were not relevant in allergic patients (processing-dependent hapten mechanism) [65,66,67]. This illustrates the need to confirm the in vivo relevance of the obtained epitope–T cell interaction, which may be shaped by low chemical concentrations in vivo, e.g., in the draining lymph nodes.

Chemical-induced epitopes may also be provided by feeding hapten-modified (self-) proteins to APC. As model carrier proteins, most studies use HSA. Within PBMC, monocytes and B cells can capture the antigen proteins and present processed peptides via MHC II to CD4+ T cells. For CD8+ T cell activation, cross-presentation and thus the use of professional APC such as MoDC is necessary [79].

### 4.3. T Cell Source

Usually, blood-derived T cells are assessed for their chemical reactivity. Only a few studies use skin-derived T cells from ACD lesions or analyze blister fluid [105,108,123,129,130]. The isolation of T cells from the skin may not be efficient and can introduce bias if antigen-specific T cells are restrained by tight immunological synapses [131,132]. Nevertheless, the frequencies of chemical-specific T cells seem increased in situ at sites of the allergic reaction [123,129,130]. Apart from the whole T cell pool, T cell subpopulations may be interrogated, e.g., CD4+ or CD8+ T cells. Magnetic enrichment or untouched depletion techniques may yield purities of ~90% or better. The required number of input T cells determines the limit of detection. Highly frequent antigen-specific T cells, e.g., nickel-specific T cells (200 µM NiSO_4_) can be detected in one well of a 96-well plate using only 0.8 × 10^6^ PBMC [39]. However, the rarer the antigen-specific T cell population is, the more T cells need to be interrogated, requiring inputs of e.g., 50–100 × 10^6^ PBMC or more. The physiological limit is the number of PBMC that can be obtained from a blood donation. Amplified T cell libraries have not yet been used in the field [133].

Conditions for T cell activation may be optimized. Besides, depletion of regulatory T cells (e.g., CD25+ T cells), addition of cytokines such as IL-12 or IL-4 or autologous serum may support the proliferation of chemical-specific T cell subsets [95,127,134,135]. In addition, the presence of co-stimulatory antibodies (e.g., α-CD28, α-CD49a) or checkpoint inhibitor antibodies (e.g., α-programmed death ligand 1/2 (PD-L1/2), α-PD-1, α-cytotoxic T-lymphocyte-associated protein (CTLA)-4) may optimize conditions for T cell activation [136].

### 4.4. Read-Outs

Proliferation-based methods such as the lymphocyte transformation test (LTT) constitute the most used read-outs for the detection of chemical-specific T cells (Table 3). Staining with pMHC multimers is not an option since chemical-induced T cell epitopes remain unknown. Besides the incorporation of radioactive nucleotides or dye dilution, proliferated T cells may also be detected by determining cytokine levels or metabolite production. Direct quantification of chemical-specific memory T cells can be accomplished ex vivo with ELISpot analysis (DNCB) (Newell, 2013) [103] or with the help of limiting dilution cultures [137]. Using LTT, the reactive T cell pool is usually not comprehensively captured since naïve T cells, for instance, proliferate only in the presence of professional APC. Original frequencies of memory T cell subpopulations will likely be lost in LTT, given the different division speeds [138,139]. Besides, ELISA results do not inform about the number of antigen-specific T cells since individual cytokine amounts secreted per cell differ. For all cytokine-based methods, a parallel analysis of several cytokines will be useful to capture different cytokine-producing subpopulations. This is of particular importance, because polarization patterns differ or have remained unclear for chemical allergens [39,140].

Activation-induced surface marker assays constitute a rather new option for a fast, comprehensive and quantitative analysis of chemical antigen-specific T cells [125,141,142]. Recently, our group adopted this technique to detect nickel-specific CD154+CD4+ naïve and memory T cells [39].

A promising emerging read-out is the analysis of chemical-specific TCR repertoires which may inform on antigen recognition mechanisms [39,143]. Bulk high-throughput sequencing may reveal peculiar gene segment use and inform on clonal expansions while single T cell clone analysis provides information on TCR α- and β-chain pairing. Flow cytometry analysis of TCR V-regions is limited by antibody availability and only informs on TRBV gene segment use. Oakes, 2017 [100] found limited V-gene segment use among ~800 PPD-specific TCR α- and β-chains, e.g., a dominant TRAV29/DV5 use, from one patient, indicating outgrowth of antigen-specific T cell clonotypes. Skazik, 2008 [101] used a panel of 24 Vβ antibodies to identify TRBV14 (Vβ16 in Arden nomenclature) expression by 5/8 PPD-specific T cell clones. Further experiments are needed to investigate the characteristics of PPD-specific TCR. For HLA-B*15:02-associated carbamazepine hypersensitivity, Ko, 2011 [144] identified an overrepresentation of TRBV25-1 (Vβ11) and TRAV9-2 (Vα22) gene segments in antigen-specific T cell lines from eight patients. Interestingly, the TRAV9-2 segment has been mechanistically linked to nickel recognition [38,39], but a connection to carbamazepine recognition remains to be shown.

### 4.5. Immune Monitoring of Allergic and Non-Allergic Individuals

For diagnostic purposes, differences in the immune responses of allergic and non-allergic individuals have to be identified. Among all chemicals investigated in the studies systematically reviewed here, the ability to detect PPD-specific T cells seems the most promising diagnostic in vitro option [79,80,93,96,99]. Mostly, studies monitor frequency differences, e.g., increased LTT stimulation indexes for allergic individuals. In general, two challenges emerge. Firstly, T cell responses may be detected only for some allergic individuals, i.e., detection levels are not sufficient to identify all allergic individuals as observed for MCI, MI and fragrance mix [95,106,116]. Secondly, frequencies of blood-derived chemical-specific T cells may be similar in allergic and non-allergic individuals, which also impedes allergy detection. BB-specific T cells are frequent in all individuals [80,93], similar to TNBS- or nickel-specific T cells. This likely occurs due to a particular interaction with a larger fraction of the TCR repertoire [39,62,137]. In such cases, allergy-associated T cell subpopulations need to be defined, which has not been accomplished yet.

Another interesting option is a TCR-based diagnosis, which has been recently accomplished for cytomegalovirus or severe acute respiratory syndrome coronavirus type 2 (SARS-CoV-2) infections [145,146]. Pan, 2019 [147] observed one public carbamazepine-specific HLA-B15:02-restricted TCR (TRBV12-4/TRBJ2-2, TCRβ CDR3 “ASSLAGELF”), which had an increased frequency in seven allergic individuals compared to 44 healthy control individuals. A pairing TCR α-chain CDR3 “VFDNTDKLI” was expressed by 83% of carbamazepine-specific TCR. However, without a known HLA association, TCR sequencing data from several hundred to thousands of individuals with defined allergy status have to be available to evaluate a TCR-based diagnostic option, an endeavor for the future when more sequences become available.

### 4.6. Possible Uses of Assays Investigating Non-Antigen-Specific T Cell Activation

Among the systematically reviewed literature, only a few studies investigated the general effects of sensitizing chemicals on T cells (Section 3.3). One reason is to investigate the T cell activation in a non-antigen-specific manner, similar to the effect that chemicals have on DC maturation or keratinocytes activation [89]. Another purpose is to study immunotoxic chemical effects, e.g., a reduction in cytokine-producing activities. In addition, mixed lymphocyte reactions serve to indicate functional chemical-induced DC maturation [88,92]. With regard to patient analysis, global changes in T cell subsets or function may be associated with the allergic state [90,91].

### 4.7. Limitations of Our Study

Our selection of original research articles focuses on a relatively small proportion of sensitizing chemicals, i.e., skin sensitizers that have been investigated by T cell assays in vitro. We focus on more recent studies published within the last 20 years. A complete assessment of all chemical allergens, including systemically acting drugs, respiratory sensitizers and additional model chemicals, would be beyond the scope of this review. However, the general findings of the present review are transferable to other sensitizing chemicals and valid in general since the in vitro setup is similar.

## 5. Conclusions and Outlook

T cell activation mechanistically underlies chemical hypersensitivity reactions. Thus, the in vitro monitoring of human T cell immune response offers a great potential.

Over the past two decades, tremendous progress has been made in the understanding of T cell epitope formation by sensitizing chemicals. Epitopes may form by various methods that are hard to predict by in silico or in chemico experiments and thus are still defined experimentally. Detected T cell responses are informative, especially if the analysis of patients illustrates in vivo relevance, while a negative result cannot be interpreted [148].

Besides pharmacologically relevant allergens, e.g., drugs, a number of skin sensitizing substances from our daily environment and some model chemicals have been successfully tested for T cell activation. The outlined experimental approaches reviewed here provide a path for the testing of additional chemicals. A broader application of new methods such as activation-induced marker assays, multi-parameter flow cytometry and high-throughput sequencing could advance the characterization of chemical-specific T cells, their phenotypes, functions and TCR characteristics [39].

A unique advantage of T cell assays is their capacity to assess cross-reactivity of individual T cell clonotypes. This can hardly be accomplished in vivo since patch testing relies on skin penetration, which differs for individual allergens and thus confounds results. In addition, prior exposure and co-sensitization cannot be ruled out in humans.

In vitro T cell assays have the potential to improve allergy diagnoses on an individual patient level, enable longitudinal tracking of immune responses, elucidate disease mechanisms and, potentially, may enable public biomonitoring in the future. T cell assays are also well-suited to complement predictive testing strategies for sensitizing chemicals in regulatory toxicology. Current in vivo tests are limited by species differences, ethical considerations and low throughput. In vitro, OECD-validated cell-based methods focus on steps prior to T cell activation, e.g., keratinocytes and DC responses, which represent interactions with the innate immune system. In the beginning era of the new approach methodologies (NAM) and next generation risk assessment (NGRA), the OECD Guideline 497 on “Defined Approaches for Skin Sensitization” has recently been published. The defined approaches currently listed combine several methods to allow hazard assessment and, in some cases, potency prediction, but lack T cell-based read-outs [149].

In summary, the specific influence of T cell activation on the sensitizing capacity of a chemical, TCR cross-reactivity and in vitro diagnostic options remain unclear until reliable T cell assays become available.

## Figures and Tables

**Table 1:** Chemical allergens showing in vitro antigen-specific T cell activation in the different reviewed studies.

*N*°	*Chemical*	*Main Use*	*Score* *	*References*
**1**	Bandrowski’s Base (BB)	**	+++	Coulter, 2010 [93]; Gibson, 2015 [94]; Moed, 2005 [95]; Sieben, 2002 [80]
**2**	*p*-Phenylenediamine (PPD)	hair dye and dye	+++	Bordignon, 2015 [96]; Coulter, 2007 [97]; Coulter, 2010 [93]; Gibson, 2015 [94]; Jenkinson, 2009 [98]; Jenkinson, 2010 [79]; Kneilling, 2009 [99]; Moed, 2005 [95]; Oakes, 2017 [100]; Sieben 2002 [80]; Skazik, 2008 [101]; Wicks, 2019 [102]
**3**	2,4-Dinitrochlorobenzene (DNCB)	model chemical	++	Betts, 2017 [78]; Newell, 2013 [103]
**4**	Balsam of Peru	fragrance	++	Nicolai, 2020 [51]
**5**	Benzyl benzoate	fragrance	++	Nicolai, 2020 [51]
**6**	Benzyl cinnamate	fragrance	++	Nicolai, 2020 [51]; Schutte, 2019 [104]
**7**	Coenzyme Q2	fragrance	++	Nicolai, 2020 [51]
**8**	Eugenol	fragrance	++	Sieben, 2001 [105]
**9**	Farnesol	fragrance	++	Nicolai, 2020 [51]
**10**	Fragrance mix	fragrance	++	Cortial, 2015 [106]; Moed, 2005 [95]
**11**	Methylchloroisothiazolinone (MCI)	preservative	++	Moed, 2005 [95]
	Methylchloroisothiazolinone/Methylisothiazolinone (MCI/MI)	preservative	++	Masjedi, 2003 [107]
**12**	Sulfamethoxazole/Trimethoprim (SMX/TMP)	drugs	++	Kim, 2020 [108]
**13**	1-Fluoro-2,4-dinitrobenzene (DNFB)	model chemical	+	Banerjee, 2003 [109]
**14**	2,4-Dinitrobenzenesulfoniacid (DNBS)	model chemical	+	Gildea, 2004 [110]
**15**	Azidamphenicol	drug	+	Sachs, 2001 [111]
**16**	Benzyl salicylate	fragrance	+	Schutte, 2019 [104]
**17**	Chloramphenicol	drug	+	Sachs, 2001 [111]
**18**	Clindamycin	drug	+	Vilchez-Sánchez, 2020 [112]
**19**	Diltiazem	drug	+	Girardi, 2005 [113]
**20**	Diphenylcyclopropenone (DPCP)	drug	+	Friedmann, 2017 [114]
**21**	Geraniol	fragrance	+	Sieben, 2001 [105]
**22**	Hydroxycitronellal	fragrance	+	Sieben, 2001 [105]
**24**	Isoeugenol	fragrance	+	Banerjee, 2003 [109]; Sieben, 2001 [105]
**23**	*Machaerium scleroxylon*	plant	+	Hansel, 2019 [115]
**24**	Methylisothiazolinone (MI)	preservative	+	Popple, 2016 [116]
**25**	Metronidazole	drug	+	Girardi, 2005 [113]
**26**	Oak moss	fragrance	+	Sieben, 2001 [105]
**27**	Parthenolide	***	+	Wahlkvist, 2008 [117]
**28**	Squaric acid dibutylester (SADBE)	drug ^$^	+	Camouse, 2008 [118]
**29**	Trichloroethylene (TCE)	pollutant	+	Li, 2019 [119]
**31**	Urushiol	***	+	Kim, 2016 [120]

* Experimental evidence for T cell activation for individual chemicals was graded according to Section 2.4 from best (+++) to little (+). ** PPD-derivative, *** plant component, ^$^ photographic revealer.

**Table 2:** APC choices and approaches for the in vitro generation of T cell epitopes used by the reviewed studies.

*APC*	*Epitope* *Formation*	*Chemicals*	*References*
*PBMC*	Direct administration in culture	Azidamphenicol, BB, Benzyl cinnamate, Benzyl salicylate, Chloramphenicol, Clindamycin, Diltiazem, DNCB, DNFB, Eugenol, Fragrance mix, Geraniol, Hydroxycitronellal, Isoeugenol, Metronidazole, *Machaerium scleroxylon*, MCI/MI, MI, Oak mos, Parthenolide, PPD, SMX/TMP, TCE	Banerjee, 2003 [109]; Bordignon, 2015 [96]; Cortial, 2015 [106]; Coulter, 2010 [93]; Friedmann, 2017 [114]; Girardi, 2005 [113]; Hansel, 2019 [115]; Jenkinson, 2009 [98]; Kim, 2020 [108]; Knelling, 2010 [99]; Li, 2019 [119]; Masjedi, 2003 [107]; Moed, 2005 [95]; Newell, 2013 [103]; Popple, 2016 [116]; Sachs, 2001 [111]; Schutte, 2019 [104]; Sieben, 2001 [105]; Sieben, 2002 [80]; Skazik, 2008 [101]; Vilchez-Sánchez, 2020 [112]; Wahlkvist, 2008 [117]; Wicks, 2019 [102]
	Modification (e.g., pulsed APC)	BB, PPD	Sieben, 2002 [80]; Wicks, 2019 [102]
	Protein conjugation (e.g., to HSA)	MI, PPD	Oakes, 2017 [100]; Popple, 2016 [116]; Wicks, 2019 [102]
*Dendritic cells*	Direct administration in culture	BB, PPD	Coulter, 2010 [93]; Gibson, 2015 [94]
	Modification (e.g., pulsed APC)	BB, DNBS, Fragrance mix, MCI, PPD, SADBE	Camouse, 2008 [118]; Coulter, 2007 [97]; Gildea, 2004 [110]; Moed, 2005 [95]
*EBV-transformed* *B cells*	Direct administration in culture	Eugenol, Geraniol, Hydroxycitronellal, Isoeugenol, Oak moss, PPD	Jenkinson, 2010 [79]; Gibson, 2015 [94]; Sieben, 2001 [105]
	Protein conjugation (e.g., to HSA)	PPD	Jenkinson, 2010 [79]
*Cell lines* *(CD1a-expressing)*	Direct administration in culture	Balsam of Peru, Benzyl benzoate, Benzyl cinnamate, Coenzyme Q2, Farnesol	Nicolai, 2020 [51]
	Modification (e.g., pulsed APC)	DNCB, Urushiol	Betts, 2017 [78]; Kim, 2016 [120]; Nicolai, 2020 [51]

EBV, Epstein Herpes Virus; HSA, Human Serum Albumin; further abbreviations are listed in Table 1.

**Table 3:** T cell activation read-outs.

*Read-outs*	*Method/Assay*	*Chemicals*	*References*
*Proliferation*	Thymidine	Azidamphenicol, ** BB, Chloramphenicol, Clindamycin, Diltiazem, DNBS, DPCP, Eugenol, ** Fragrance mix, ** Geraniol, ** Hydroxycitronellal, ** Isoeugenol, ** MCI, ** MCI/MI, ** MI, Metronidazole, ** Oak moss, ** PPD, SADBE	Camouse, 2008 [118]; ** Cortial, 2015 [106]; Coulter, 2007 [97]; ** Coulter, 2010 [93]; Friedmann, 2017 [114]; ** Gibson, 2015 [94]; Gildea, 2004 [110]; Girardi, 2005 [113]; Jenkinson, 2009 [98]; ** Jenkinson, 2010 [79]; Kneilling, 2010 [99]; ** Masjedi, 2003 [107]; ** Moed, 2005 [95]; Oakes, 2017 [100]; Popple, 2016 [116]; Sachs, 2001 [111]; ** Sieben, 2001 [105]; ** Sieben, 2002 [80]; Skazik, 2008 [101]; Vilchez-Sánchez, 2020 [112]; ** Wicks, 2016 [102]
	CFSE	*Machaerium scleroxylon*, SMX/TMP	Kim, 2020 [108]; Hansel, 2019 [115]
	Other	Benzyl cinnamate, ** Benzyl salicylate, ** DNFB, DPCP, ** Isoeugenol, ** TCE	** Banerjee, 2003 [109]; Friedmann, 2017 [114]; ** Li, 2019 [119]; ** Schutte, 2019 [104]
*Cytokine* *production*	ELISA	Balsam of Peru, ** BB, Benzyl benzoate, Benzyl cinnamate, Coenzyme Q2, DNCB, ** DNFB, Eugenol, Farnesol, ** Fragrance mix, ** Geraniol, ** Hydroxycitronellal, ** Isoeugenol, ** MCI, ** MI, ** Oak moss, ** PPD, ** TCE	Banerjee, 2003 [109]; Betts, 2017 [78]; ** Cortial, 2015 [106]; ** Coulter, 2010 [93]; ** Jenkinson, 2010 [79]; ** Li, 2019 [119]; ** Masjedi, 2003 [107]; ** Moed, 2005 [95]; Nicolai, 2020 [51]; ** Sieben, 2001 [105]; ** Sieben, 2002 [80]
	ELISpot	** Benzyl salicylate DNCB, PPD, Parthenolide	Bordignon, 2015 [96]; Gibson, 2015 [94]; Newell, 2013 [103]; ** Schutte, 2019 [104]; Wahlkvist, 2008 [117]
	Other	DNCB, Urushiol	Betts, 2017 [78]; Newell, 2013 [103]; Kim 2016 [120]
*Gene expression*	RT-PCR	BB, PPD, Urushiol	Coulter, 2010 [93]; Kim, 2016 [120]
	Microarray/RNA seq	DNBS, SMX/TMP	Gildea, 2004 [110]; Kim, 2020 [108]
*T cell phenotype* *(e.g., activation markers, cytotoxicity)*		BB, DNCB, Eugenol, Geraniol, Hydroxycitronellal, Isoeugenol, *Machaerium scleroxylon*, Oak moss, PPD, SMX/TMP, TCE	Hansel, 2019 [115]; Kim, 2020 [108]; Li, 2019 [119]; Sieben, 2001 [105]; Sieben, 2002 [80]; Wicks, 2019 [102]
*T cell clone* *Proliferation*	w/o HLA blocking	PPD	Gibson, 2015 [94]; Jenkinson, 2010 [79]; Skazik, 2008 [101]
	with HLA blocking	BB, PPD	Sieben, 2002 [80]
*T cell receptor* *repertoire*	NGS	PPD	Oakes, 2017 [100]
	other	PPD	Skazik, 2008 [101]

** Chemical ability to induce both proliferation and cytokine secretion was measured. CFSE, carboxy fluorescein diacetate succinimidyl ester; HLA, human leukocyte antigen; ELISA, enzyme-linked immuno-sorbent assay; ELISpot, enzyme-linked immuno-spot; NGS, next generation sequencing; RT-PCR, real-time quantitative polymerase chain reaction; further abbreviations are listed in Table 1.

## Supplementary Materials

The following are available online at https://www.mdpi.com/article/10.3390/cells11010083/s1, Table S1: Pubmed search results, Table S2: Chemicals allergens showing in vitro antigen-specific T cell activation in the different reviewed studies (extension of Table 1), Table S3: Studies describing general effects of non-metallic chemical allergens on T cell function.

## Abbreviations

ACD: allergic contact dermatitis; APC, antigen-presenting cell; CDR, complementarity-determining region; DC, dendritic cell; HSA, human serum albumin; HLA, human leukocyte antigen; LTT, lymphocyte transformation test; MHC, major histocompatibility complex; PBMC, peripheral blood mononuclear cells; p-i, pharmacological interaction; TCR, T cell receptor.
