# Supplementary material for: In Vitro Monitoring of Human T Cell Responses to Skin Sensitizing Chemicals—A Systematic Review
Source: Cells. 2021 Dec 28;11(1):83. doi: 10.3390/cells11010083 (PMC8750770; doi:10.3390/cells11010083)
Supplement: Supplementary file 1 [file cells-11-00083-s001.zip › Table S1.pdf]

Table S1. Pubmed search results.

#	Search History	Results
#1	("t-lymphocytes"[MeSH Terms] AND ("2001/01/01 00:00":"3000/01/01 05:00"[Date - Publication] AND "journal article"[Publication Type] NOT review[Filter]))	169,883
#2	(("dermatitis, allergic contact"[MeSH Terms] OR "chemical allergen"[Title/Abstract] OR "chemical allergens"[Title/Abstract] OR ("hypersensitivity"[Title/Abstract] AND "dermatitis"[Title/Abstract])) AND ("2001/01/01 00:00":"3000/01/01 05:00"[Date - Publication] AND "journal article"[Publication Type] NOT review[Filter]))	6,748
#3	#1 AND #2	445
#4	#3 AND English[lang]	429
#5	#4 AND (human[All Fields])	238
