# Supplementary material for: In Vitro Monitoring of Human T Cell Responses to Skin Sensitizing Chemicals—A Systematic Review
Source: Cells. 2021 Dec 28;11(1):83. doi: 10.3390/cells11010083 (PMC8750770; doi:10.3390/cells11010083)
Supplement: Supplementary file 1 [file cells-11-00083-s001.zip › Table S3.pdf]

**Table S3.** Studies describing general effects of non-metalic chemical allergens on T cell function.

| References | Chemicals | Read-out | Experimental system | Patients | Remarks |
|---|---|---|---|---|---|
| **Hou, 2020 [79]** | 2-Mercaptobenzothiazole<br>2-Methoxy-4-methylphenol<br>4-Methylaminophenol sulfate<br>4-Nitrobenzyl bromide<br>Benzo[a]pyrene<br>Cyclamen aldehyde<br>Diethyl maleate<br>DNCB<br>Ethyl acrylate<br>Eugenol<br>Formaldehyde<br>Glutaraldehyde<br>Glyoxal<br>Hexyl cinnamic aldehyde<br>Hydroxycitronellal<br>Imidazolidinyl urea<br>Linalool<br>m-Aminophenol<br>p-Benzoquinone<br>Phenyl benzoate<br>PPD<br>Resorcinol<br>Tetramethylthiuram disulfide<br>trans-Cinnamaldehyde | CD69 relative fluorescence intensity | Jurkat clone E6-1<br>human T lymphocyte cell line | no | Cell viability >50% confirmed<br>Jurkat T cells only express one TCR<br>CD69 as T cell activation marker can be upregulated TCR-independently (Frentsch et al., 2005) |
| **Clouet, 2019 [82]** | Cinnamaldehyde<br>Eugenol<br>Farnesol<br>Geraniol<br>MI<br>Resorcinol | T cell proliferation detected by CSFE dilution | THP-1 cell line<br>allogeneic CD4+ T cells from PBMC coculture | no | Mixed lymphocyte reaction (non-autologous system) |
| **Frombach, 2018 [78]** | 2-Mercaptobenzothiazole<br>DNCB<br>PPD | IL-23R /CD119, CD44 and CD124 expression<br>IL-4, IL-17 and IFN-$\gamma$ secretion | Primary human keratinocytes, moDCs and non-autologous lymphocytes from PBMC coculture | no | Mixed lymphocyte reaction (non-autologous system) |

| | | | | | |
|---|---|---|---|---|---|
| **Mai, 2017 [81]** | Formaldehyde | IL-17 and IL-22 expression and secretion | Fresh heparinized blood incubated with PMA-Ionomycin | 104 workers exposed to low-level formaldehyde (19/104 with history of ACD)<br>90 unexposed controls | Immunophenotyping of PMA-Ionomycin stimulated T cells reveals differences in T cell subsets<br>No antigen-specificity assessed |
| **Baló-Banga, 2015 [80]** | Clindamycin | IL-6 secretion | IL-6 solid phase immunoassay in PBMC | Analysis of heterogeneous patient groups by different in vivo diagnostic tests to complement in vitro testing<br>Specific results of IL-6 release only for one patient | 20 minutes drug incubation of PBMC in tubes (suspension culture): little direct contact expected between T cells and potential APC |

ACD, allergic contact dermatitis; APC, antigen presenting cells; CD, cluster of differentiation; CSFE, carboxyfluorescein diacetate succinimidyl ester; DNCB, 2,4-Dinitrochlorobenzene; DNFB, 1-Fluoro-2,4-dinitrobenzene; IFN, interferon; IL, interleukin; MI, Methylisothiazolinone; moDCs, monocytes-derived dendritic cells; PBMC, peripheral blood mononuclear cells; PMA, phytohemagglutinin; PPD, *p*-Phenylenediamine; TCR, T cell receptor.

Additional reference:

Frentsch, M., Arbach, O., Kirchhoff, D., Moewes, B., Worm, M., Rothe, M., Scheffold, A., and Thiel, A. (2005). Direct access to CD4+ T cells specific for defined antigens according to CD154 expression. Nat Med *11*, 1118-1124.
